# 

**Published:** 1890-07

**Authors:** 


					﻿Vol. X.
JULY, 1890.
NO. 7.
UHE
fiOMEOPATHlG pHY^lClAfJ,
A Monthly Journal of Homoeopathic Materia Medica
and Clinical Medicine.
“ He is a freeman whom truth makes free, and, all are slaves beside.”
“Seek the Truth: come whence it may, cost what it witL."
EDITED AND PUBLISHED BY
WALTER ML JAMES, M-. E>.,
AND
GEORGE H. CLARK, M. D.
ASSISTED BY THE FOLLOWING CONTRIBUTORS:
E. T. Balch, M. D., South Bend, Wash.
Clarence Willard*Butler, m. D.,
Montclair, N. J.
Prof. Edmund Carleton, M. D., New
York.
Daniel W. Clausen, M. D., Philada.
Stuart Close, M.D., Brooklyn.
Edward Cranch, M. D., Erie, Pa.
W. S. Gee, M. D.. Hyde Park, Ill.
Clarence C. Howard, M.D., New York.
Samuel A. Kimball, M. D., Boston.
Edmund J. Lee, M. D., Former Editor,
Philada.
A. McNeil, M. D., San Francisco.
C. F. Nichols, M. D., Boston.
Mahlon Preston, M. D., Norristown,
George W. Sherbino, M. D., Abilene,
Texas.
C. Carleton Smith, M. D., Fhilada.
Wm. Stein kauf, M. D., St. Charles, Mo.
PHILADELPHIA:
ZNöLllJaS SBJtTTCID STRHJBJ'T,
X.Q 2ST Z> O 3T
ALFRED HEATH & CO., Sole Agents FOR Gr^at Britain,
114 Ebury Street, Eatou Square.
XJSJ'ITeari// Subscription, $2,50, invariably in advance. Single number», 25 cts.
Entered at the Philadelphia Poet-Office ae Second-Claw Mall Matter.
				

